# Invasive‐plant traits, native‐plant traits, and their divergences as invasion factors

**DOI:** 10.1002/ece3.11525

**Published:** 2024-06-25

**Authors:** Yang Li, Ming Yue, Yuchao Wang, Zhuxin Mao, Jinlin Lyv, Qian Li

**Affiliations:** ^1^ Xi'an Botanical Garden of Shaanxi Province Institute of Botany of Shaanxi Province Xi'an China; ^2^ Shaanxi Engineering Research Centre for Conservation and Utilization of Botanical Resources Xi'an China; ^3^ Shaanxi Key Laboratory of Qinling Ecological Security Shaanxi Institute of Zoology Xi'an China

**Keywords:** biotic resistance, competition, invasiveness, phenotypic divergence hypothesis

## Abstract

Invasive plants exert significant ecological impacts on native plants, communities, and ecosystems. However, consistent conclusions regarding how traits of invasive plants, native plants, and their divergences affect invasion dynamics are still lacking. Here, we conducted a pairwise common garden experiment to investigate how invasion was influenced not only by invasive plants but also by native plants, aiming to elucidate the role of invasive‐plant traits, native‐plant traits, and their divergences in invasion processes. Our findings revealed variations in invasive stage depending on the combinations of invasive and native plants. Specifically, native plants such as *A. argyi*, *A. lavandulifolia*, and *C. album* exhibited competitive superiority when co‐occurring with the three invasive plants. *S. viridis*, *A. vestita*, and *A. annua* had competitive superiority when they co‐occurred with *E. canadensis*, *G. quadriradiata*, and *E. annuus* respectively. Furthermore, our results demonstrated that the competitive abilities of invasive plants were primarily influenced by factors such as height, diameter, and biomass allocation, while native plants' competitive abilities were mainly affected by diameter, biomass allocation, and function group differences. Moreover, our analysis revealed that invasive‐plant traits, native‐plant traits, their divergences, and their interactions together explained 36.88% of the variation in invasion dynamics, with invasive‐plant traits and the native‐plant traits explaining 10.19% and 6.88%, respectively. In conclusion, the traits of invasive and native plants, along with their divergences, significantly influence interspecific relationships, and influencing the invasive stages. Divergences in competitive strategies between the native plants and invasive plants facilitated invasion processes. Our study not only contributes to understanding the mechanisms underlying invasion, but also provides a scientific foundation for predicting and managing the negative effects of invasive plants.

## Graphical abstract

The interspecific relationships between 3 invasive plants and 10 native plants. >0 meant that the native plant took competitive superiority in the mixture, while <0 meant the invasive plant took competitive superiority in the mixture.

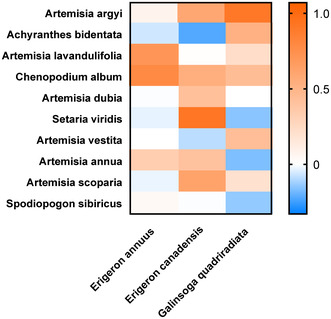

## 1 INTRODUCTION

Plant invasion is a major environmental problem, posing a major threat to native ecosystems (Seebens et al., 2018; Sheppard & Brendel, 2021; Vilà et al., 2011). Thus, one of the main aims of invasion biology research was to determine and predict the impacts of invasive plants on native communities.

Theoretical and empirical studies have confirmed that successful invasion depends on invasive plants and native plants (Vilà & Weiner, 2004). Some studies have suggested that there may be some traits related to invasiveness, because invasive plant species are not random samples of all plants worldwide (Divíšek et al., 2018). Traits associated with competitiveness are generally considered to be associated with invasiveness (Chen et al., 2018; Van Kleunen et al., 2010; Whitney & Gabler, 2008). However, traits that allow an invasive plants to successfully establish in certain habitats and persist in plant communities may not be advantageous in other habitats (Divíšek et al., 2018; Theoharides & Dukes, 2007). It implies that the native plants may also influence invasion. Moreover, the phenotypic divergence hypothesis predicts that an introduced species would invade successfully because the recipient community lacks ecologically similar species (Divíšek et al., 2018; Ordonez et al., 2010). In contrast, the phenotypic convergence hypothesis predicts that an introduced species would invade successfully because it could share similar traits with native species (Divíšek et al., 2018; Ordonez et al., 2010). Studies also found that a plant needs to be similar enough to be admissible to a native community when first introduced, while it also needs to be different enough to exclude other species after successful colonization (Ordonez et al., 2010). These studies indicated that invasive stage relied on the invasive plant, the native plant, as well as their divergences.

Quantifying the strength of interactions between native and invasive plants is a key approach in invasion biology (Bowler et al., 2022). Competition, as a form of biotic selection, can trigger eco‐evolutionary shifts between invasive and native plants when invasive plants are introduced (Germain et al., 2020; Lankau, 2011; Parré et al., 2023; Sheppard & Brendel, 2021; Sheppard & Schurr, 2019), ultimately shaping the invasive outcome. Invasive plants must compete for limited resources with the native plants in the recipient community, and the native plants may have developed niche differentiation and reached a balance in competitive abilities through adaptive evolution (Thorpe et al., 2011). In the process of invasion, invasive plants can adapt to competition with native plants and maintain competitive superiority. In contrast, native plants could resist invasive plants during the process of competition and co‐evolution with invasive plants in the recipient community (Sheppard & Brendel, 2021). This dynamic suggested a reciprocal inhibition between invasive and native plants, where each could impede the other's success. However, according to the empty niche hypothesis, invasive plant success is greater if it could take advantage of resources that are not available to native plants in the recipient community (Mitchell et al., 2006). This implied that interspecific interactions between invasive and native plants could range from positive to neutral (Bowler et al., 2022). Plant invasion is an ongoing process and can be quantified by comparing the ratios of invasive to native plants (Ren et al., 2022). Consequently, evaluating the relative performance of invasive and native plants in competition is essential for assessing the degree of invasion.

The Qinling Mountains are widely recognized as one of the important biodiversity hotspots in China (Yu et al., 2023). It plays important ecological functions of regulating climate, conserving soil and water, and maintaining biodiversity. However, as urbanization and economic developed rapidly, urban areas and populations continued to expand, bringing the main urban area closer to the Qinling Mountains. This proximity accelerates the spread and expansion of invasive plants (Zhang et al., 2017). The three invasive plants that we studied, *Erigeron annuus*, *E. canadensis*, and *Galinsoga quadriradiata*, are widely distributed in the Qinling Mountains according to our previous research (Lyv et al., 2023). Furthermore, we found that the three invasive plants were in different invasive stages in different recipient communities in the Qinling Mountains. Therefore, we speculated that the invasive stage was also closely related to native plants. Moreover, we wanted to identify which traits of invasive plants and native plants could determine invasive stage in the Qinling Mountains. Here, we selected 10 native dominant species which often present with the three invasive plants in the Qinling Mountains. We conducted a common garden experiment including the 3 invasive plant species and the 10 native plant species to test and identify: (1) whether the three widely distributed invasive plants both have better competitive ability than the dominant native plants in recipient communities in the Qinling Mountains? (2) Which traits of the invasive plant and the native plant, or their divergences would contribute to invasion? The purpose of our study was to understand the mechanisms of invasion and provide scientific advices for predicting and managing the negative impacts of invasive plants.

## 2 METHODS

### 2.1 Study species

We selected 3 invasive plant species and 10 native plant species. The three invasive plants were *Erigeron annuus*, *E. canadensis*, *and Galinsoga quadriradiata*, respectively. The 10 native dominant plants were *Artemisia argyi*, *Achyranthes bidentata*, *A. lavandulifolia*, *Chenopodium album*, *A. dubia*, *Setaria viridis*, *A. vestita*, *A. annua*, *A. scoparia*, and *Spodiopogon sibiricus*, respectively. The three invasive plants were selected because of their wide distribution in China (Ma, 2020). The 10 native plants were selected because they often present with the three invasive plants in the wild and exhibit different growth performances (Shou et al., 2014; Wang et al., 2022).

### 2.2 Experimental set‐up

We conducted a pairwise competition experiment at a field station in the Xi'an Botanical Garden in Shaanxi Province, China (36°46′ N, 109°12′ E). Seeds of the 3 invasive plants and the 10 native plants were collected from the Qinling Mountains, China. All the seeds we used in the study were collected from the Qinling Mountains. In 2020, the seeds of each plant were collected separately at the time of seed maturity.

The experimental design was a completely randomized design with 3 invasive species and 10 native species. Each invasive plant grew with each native plant. There were two seedlings per pot: one invasive plant and one native plant. There were 30 native–invasive combinations. Additionally, all 13 species, invasive plants and native's plants, grew in pots under intraspecific competition. We used the ratio of the performance of intra‐ and interspecific competition to assess the competitiveness. Experiments were performed in triplicate. In total, we established 129 pots, of which the 39 pots with intraspecific competition, as well as 90 pots in the interspecific competition. Two plants from each pot were placed approximately 8 cm apart. We used 129 pots with a volume of 10 L (24 cm diameter, 21 cm height) filled with field soil. All pots were placed in the open air and watered sufficiently using a drip watering system. The soil in the pots was checked every day to make sure it was moist. The pots were repositioned every 10 days to avoid environmental heterogeneity.

Soil was collected from communities where the invasive plants and the native plants present together. Soil was thoroughly stirred and then filled with pots and trays in this experiment. Seeds were germinated in trays in a greenhouse on March 10, 2021. The invasive seedlings have grown to about 3–4 cm, and the native seedlings have grown to about 3–5 cm by April 1, 2021. For each species, the seedlings with the best growth and similar height were selected. The seedlings were transplanted into pots on April 15, 2021, according to the design described. All the plants were harvested on September 28, 2021.

### 2.3 Trait selection and data collection

We aimed to develop a method to quickly determine whether an invasive plant poses a hazard to a local community. To make our results more broadly applicable, we chose morphological traits such as plant height and diameter, as well as plant functional groups, rather than typical functional traits such as specific leaf area, leaf mass ratio, leaf nitrogen content, and photosynthetic rate. It is because morphological traits can be easily obtained from most databases (Sheppard, 2018), as well as during field surveys. In the study, we divided plants into three functional groups: grass, forbs, and legumes.

The height of each individual plant and the base diameter of the main trunk or cluster were measured before harvest. After the measurements, the plants were pulled out, and the soil was shaken as much as possible. After the two plants were separated, the roots were rinsed with water. The roots left in the soil were carefully picked out and rinsed with water. In the mixed pots, it was easy to distinguish roots from each other because of the different characteristics of the different plant roots. In the monoculture pots, the remaining roots were equally divided. Each plant was divided into aboveground and belowground components. All the aboveground and belowground components were put in envelopes and numbered them. No plants produced seeds during the experiment. We dried them at 70°C for 72 h to obtain constant weights. We then measured the weight of the aboveground and belowground parts and calculated whole‐plant biomass for each plant.

### 2.4 Data analysis

Interspecific relationships are commonly thought to underlie plant invasions. A strong ability to invade is considered to be a high competitive ability of invasive plants (Sheppard, 2018), whereas a strong ability to resist invasion is considered to be a high competitive ability of native plants (Ferenc & Sheppard, 2020). Successful invasion is not only related to invasive ability but also to invasive impacts (Sheppard & Brendel, 2021); therefore, the competitive abilities of invasive plants and native plants were both considered in our study. We considered the relative performance of the mixture to represent competitive ability. The relative performances of the mixtures were calculated as the log‐response ratio (LnRR).

$$\text{LnRR}=\ln\frac{B_{\mathrm{mix}}}{B_{\text{mono}}}$$

where *B*_mix_ is the biomass of the target plant in the mixture and *B*_mono_ is the mean performance of target individuals in intraspecific competition (Sheppard, 2018).

In this study, the LnRR of biomass (referred to here as “LnRR biomass”) served as a measure of the relative ability to tolerate heterospecific neighbors, with positive values indicating a greater capacity for intraspecific competition and negative values indicating a higher level of interspecific competition. For each mixed‐plant plot, we calculated the LnRR biomass for both invasive and native plants. Positive LnRR biomass values suggested that the plant was better at tolerating heterospecific neighbors than that of the same species, whereas negative values indicated a higher tolerance for conspecific neighbors. Moreover, we calculated the difference in LnRR biomass between native and invasive plants within each pot. By averaging these values over three replicates with the same combination of invasive and native plants, we captured the interspecific relationships. Positive differences signified an advantage for native plants in competition, whereas negative differences suggested an advantage for invasive plants.

One mixed model with invasive species identity as a random effect was conducted to test the effects of native species on LnRR biomass of invasive plants. One mixed model with native species identity as a random effect was conducted to test the effects of invasive species on LnRR biomass of native plants.

We also measured and calculated the invasive‐plant traits, the native‐plant traits, and their divergences. The traits of plants included height, diameter, and biomass allocation (height of invasive plant: IH, diameter of invasive plant: ID, the ratio of root biomass to shoot biomass of invasive plant: IRS; height of native plant: NH, diameter of native plant: ID, the ratio of root biomass to shoot biomass of native plant: NRS). In addition to the differences in height and biomass between native and invasive plants at the individual level per pot (the divergence of height between native and invasive plants: H‐H, the divergence of biomass between native and invasive plants: B‐B), the functional group differences (the divergence of function type between native and invasive plants: F‐F) were also listed as divergences between the invasive and native plants in our study. The three invasive plants all belonged to forb, while the 10 native plants belonged to forb and grass, respectively. If two heterospecific plants in a pot belonged to the same functional group, the F‐F was coded by a value of 1; otherwise, the F‐F was coded by a value of 2. The divergence in height or biomass was defined as follows:

$$\left|X\right|=\left\{\begin{array}{c} -x,x<0\\ x,x\geq 0 \end{array}\right.$$

where *X* is the difference in height or biomass between native and invasive plants in per pot.

Multiple linear regression analysis with the enter method was performed to quantify the contribution of the invasive‐plant traits, the native‐plant traits, and their divergences to the LnRR biomass of invasive or native plants, respectively.

We conducted a redundancy analysis (RDA), with LnRR biomass of invasive and native plant as response variables, and the invasive‐plant traits, the native‐plant traits, and their divergences as explanatory variables, to comprehensively analyze the leading factors affecting invasion. To avoid multicollinearity and overfitting, the variable using a forward selection method was selected before analysis. Each variable was standardized to have a mean of zero and a standard deviation of one.

Then, with the invasive‐plant traits, the native‐plant traits, and their divergences as explanatory variables, variance partitioning analysis (VPA) was used with the vegan package in the R software to test the degree to which different types of factors and their combinations explained the changes in the LnRR biomass of invasive and native plants. All the above analyses were conducted using the SAS software 9.3 (SAS Institute Inc., Cary, NC, USA) and R program 4.3.2 Venn diagram was drawn by Microsoft Office 2016. Other figures were created using GraphPad Prism.

## 3 RESULTS

### 3.1 The competitive abilities of invasive plants and native plants

**FIGURE 1:**
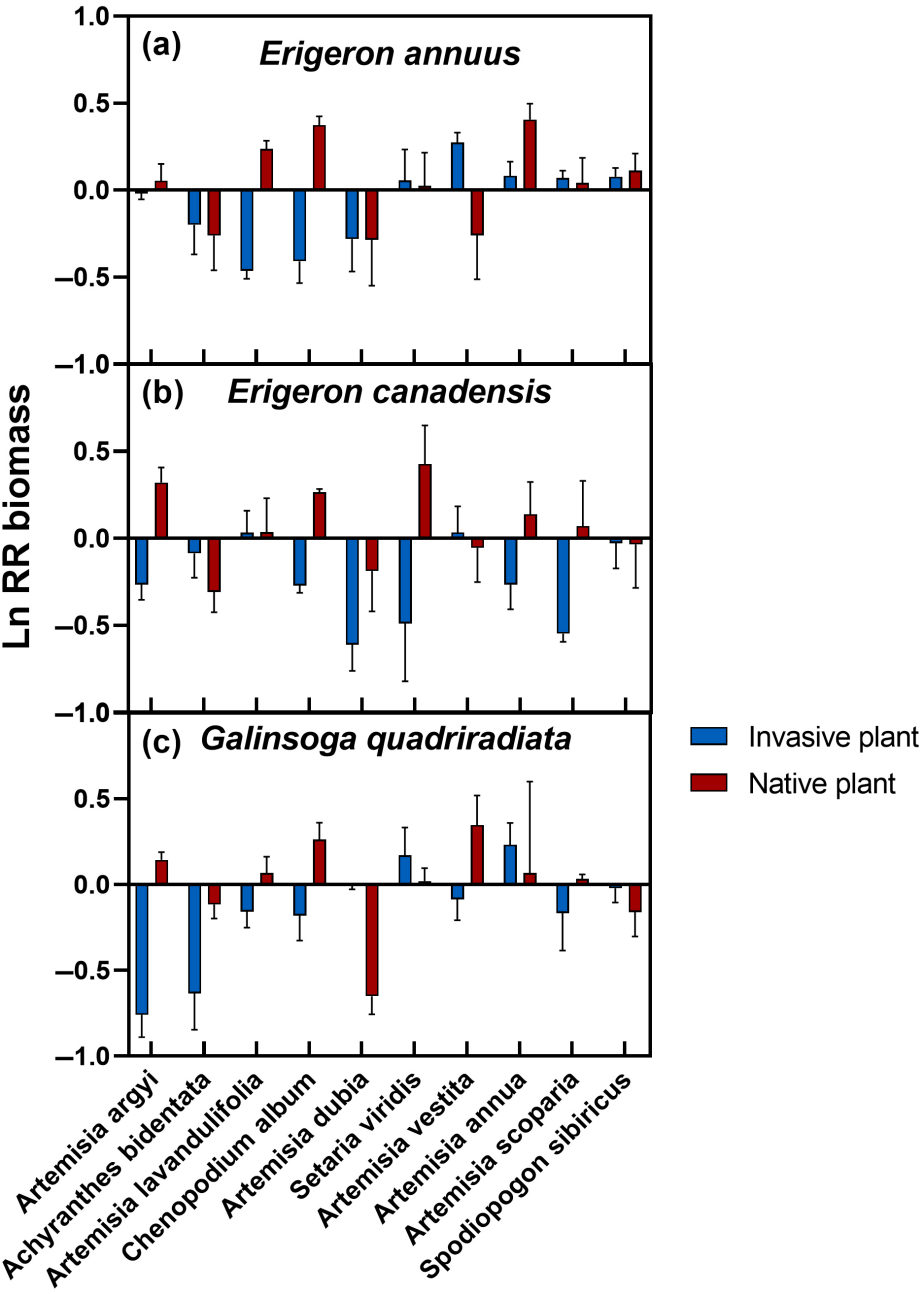
LnRR biomass of the 3 invasive plants and the 10 native plants when they mixed. Values were Mean ± SE.

The three invasive species differed in LnRR biomass in general. Across all pots, the mean LnRR biomass of the invasive plant was −0.16 ± 0.03 (*n* = 90), of which the mean LnRR biomass of *E. annuus* was −0.08 ± 0.05 (*n* = 30), the mean LnRR biomass of *G. quadriradiata* was −0.16 ± 0.07 (*n* = 30), and the mean LnRR biomass of *E. canadensis* was −0.25 ± 0.06 (*n* = 30), respectively. The LnRR biomass of the invasive plants was different from the 10 native competitors. The results of one mixed model with invasive species identity as a random effect showed that native species had significant effects on the LnRR biomass of the invasive plant (*p* = .03). For *E. annuus*, the LnRR biomass was higher than 0 when mixed with *S. viridis*, *A. vestita*, *A. annua*, *A. scoparia*, and *S. sibiricus*, respectively (Figure 1a). For *E. canadensis*, the LnRR biomass was higher than 0 when mixed with *A. lavandulifolia* and *A. vestita*, respectively (Figure 1b). For *G. quadriradiata*, the LnRR biomass was higher than 0 when mixed with *S. viridis* and *A. annua*, respectively (Figure 1c).

Furthermore, the three native species differed in their LnRR biomass in general. And the results of one mixed model with native species identity as random effect showed that invasive species did not influence significantly the LnRR biomass of the native plant (*p* > .05). The LnRR biomass of *A. bidentata* and *A. dubia* were less than 0 when mixed with three invasive plants, respectively (Figure 1a–c). The LnRR biomass of *A. vestita* was less than 0 when mixed with *E. annuus* and *E. canadensis*, respectively (Figure 1a,b). The LnRR biomass of *S. sibiricus* was less than 0 when mixed with *E. canadensis* and *G. quadriradiata*, respectively (Figure 1b,c).

**FIGURE 2:**
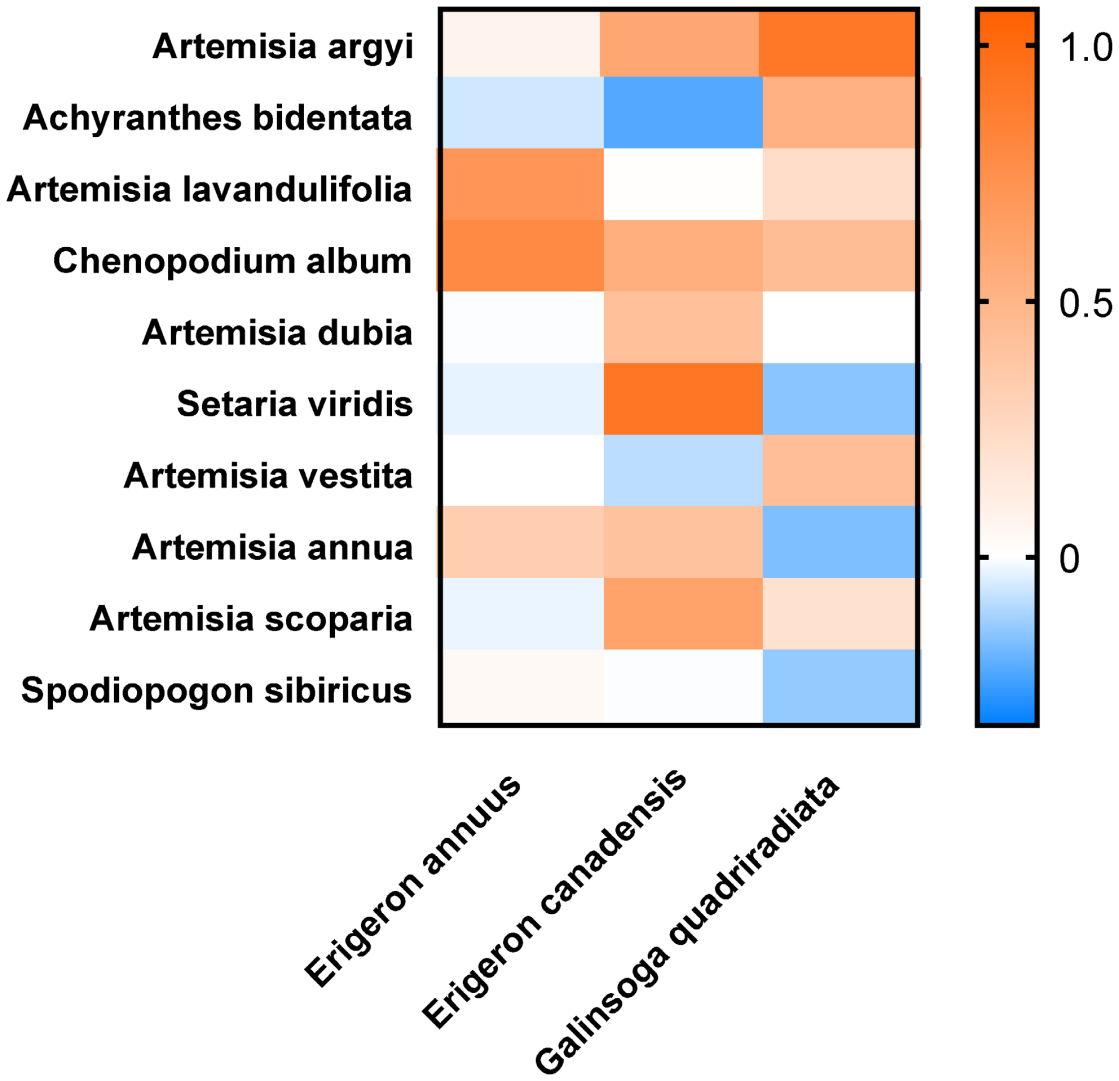
Interspecific relationships between 3 invasive plants and 10 native plants. Horizontal coordinates: the three invasive plants; vertical coordinates: the 10 native plants. The numbers represented the competitive outcome between invasive and native plants, and were calculated by the differences in the LnRR biomass of the native plant and the invasive plant in each of native–invasive combinations. Value >0 meant that the native plant took competitive superiority in the mixture, while <0 meant the invasive plant took competitive superiority in the mixture.

The interspecific relationships between invasive plants and native plants were species‐specific (Figure 2). Our results showed that invasive plant *E. annuus* may take competitive advantages when it mixed with *A. bidentata*; invasive plant *E. Canadensis* may take competitive advantages when it mixed in the pots with *A. bidentata*, and *A. vestita*; invasive plant *G. quadriradiata* may take competitive advantages when it mixed in the pots with *S. viridis*, *A. annua*, and *S. sibiricus* (Figure 2). Our results also showed that the native species such as *A. lavandulifolia*, *C. ablum*, and *A. annua* could inhibit the invasion of *E. annuus*; native species such as *A. argyi*, *C. ablum*, *A. dubia*, *S. viridis*, *A. annua*, and *A. scoparia* could inhibit the invasion of *E. Canadensis*; native species such as *A. argyi*, *A. bidentata*, *A. lavandulifolia*, *C. ablum*, *A. vestita*, and *A. scoparia* could inhibit the invasion of *G. quadriradiata* (Figure 2).

### 3.2 Factors affecting the competitive ability of invasive plants and native plants

The results of the multiple linear regression analysis indicated significant effects of the invasive plant height, diameter, and biomass allocation on the LnRR biomass of the invasive plant (all *p* < .05, Table 1). Specifically, an increase in invasive plant height, diameter, and aboveground biomass allocation correlated with an increase in the LnRR biomass of the invasive plant. Moreover, native plant height and diameter exhibited marginal effects on the LnRR biomass of the invasive plant (both *p* < .10, Table 1). The LnRR biomass of the invasive plant tended to increase with decreasing native plant height and the increasing of native plant diameter. Furthermore, the multiple linear regression analysis revealed significant effects of native plant diameter, biomass allocation, and functional group divergence on the LnRR biomass of the native plant (all *p* < .05, Table 1). The LnRR biomass of the native plant increased with increasing plant diameter and root biomass allocation. Additionally, a significant increase in LnRR biomass of the native plant was observed when native and invasive plants belonged to different functional groups.

**TABLE 1:** Effects of invasive‐plant traits, native‐plant traits, and their divergences on the LnRR biomass of invasive plants and native plants, respectively.

	LnRR biomass of invasive plant		LnRR biomass of native plant	
	Standardized regression coefficients	*p*	Standardized regression coefficients	*p*
Invasive‐plant traits				
IH	.371	**<.001**	.149	.148
ID	.590	**<.001**	−.018	.862
IRS	−.170	.052	−.058	.530
Native‐plant traits				
NH	−.266	.075	.135	.393
ND	.211	.068	.369	**.003**
NRS	−.061	.502	−.288	**.004**
The divergences				
F‐F	.139	1.460	.253	**.014**
B‐B	−.201	1.040	.067	.616
H‐H	.073	.560	.026	.845

*Note*: Bold numbers in the column indicated *p* < .05.

Abbreviations: B‐B, the divergence in biomass; F‐F, the divergence in functional type; H‐H, the divergence in height; ID, invasive plant diameter; IH, invasive plant height; IRS, the ratio of root biomass to shoot biomass for invasive plant; ND, native plant diameter; NH, native plant height; NRS, the ratio of root biomass to shoot biomass for native plant.

**FIGURE 3:**
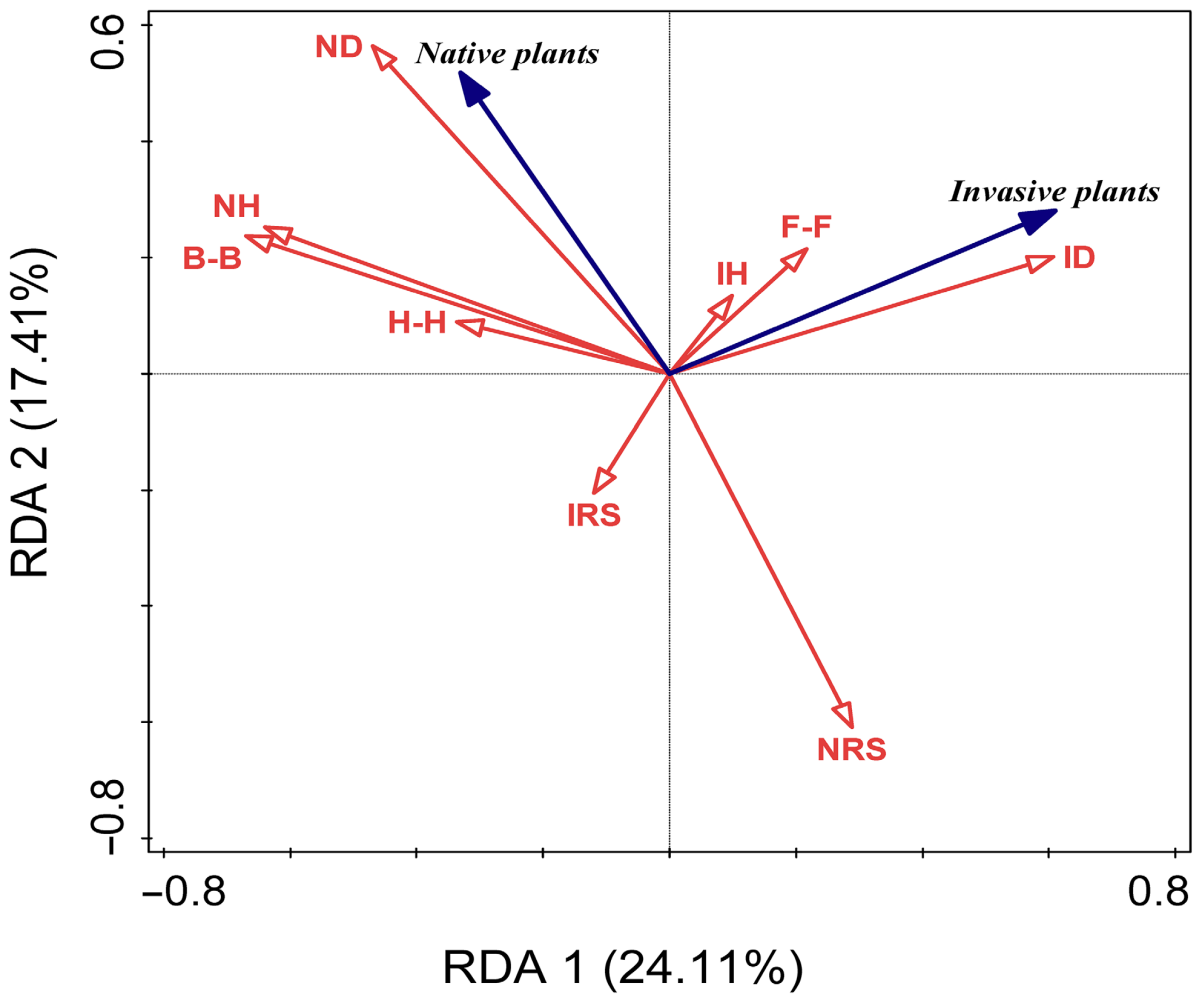
Redundancy analysis (RDA) ordination diagram displaying the major drivers of invasion. Response variables: Invasive plant LnRR biomass of invasive species; Native plant LnRR biomass of native species. Explanatory variables: IH: invasive plant height; ID: invasive plant diameter; IRS: the ratio of root biomass to shoot biomass for invasive plant; NH: native plant height; ND: native plant diameter; NRS: the ratio of root biomass to shoot biomass for native plant; F‐F: the divergence in functional type; B‐B: the divergence in biomass; H‐H: the divergence in height.

RDA results showed that the RDA1 and RDA2 axes explained 24.11% and 17.41% of the total variation, respectively, and they together explained 41.50% of the total variation in competition (*F* = 6.3, *p* < .05, Figure 3). Invasive plant height (IH), diameter (ID), and functional type divergence (F‐F) were positively correlated with the LnRR biomass of the invasive plant, while, invasive plant R/S ratio (IRS), the divergences of height and biomass (H‐H, B‐B), and the native plant height (NH) were negatively correlated with the LnRR biomass of the invasive plant. Native‐plant traits (ND, NH), and the divergences (B‐B, H‐H) were positively correlated with the LnRR biomass of the native plant (Figure 3).

### 3.3 The relative contributions of different factors to invasion

**FIGURE 4:**
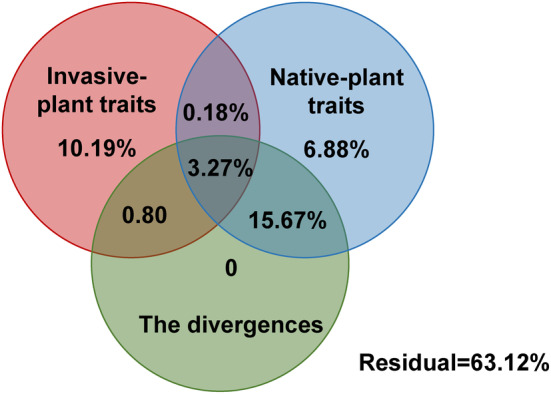
Variation partitioning in invasion. Invasive‐plant traits: plant height, plant diameter, and the ratio of root biomass to shoot biomass for invasive plant; native‐plant traits: plant height, plant diameter, and the ratio of root biomass to shoot biomass for native plant; the divergences: divergences in functional type, biomass and plant height between the invasive plants and the native plants.

VPA results revealed that the invasive‐plant traits, the native‐plant traits, the interaction between the native‐plant traits and the divergences, the interaction among the invasive‐plant traits, the native‐plant traits, and the divergences explained 10.19%, 6.88%, 15.67%, and 3.27% of the variation in invasion, respectively (Figure 4).

## 4 DISCUSSION

Our finding revealed general differences in LnRR biomass among the three invasive plants and their response to the 10 native competitors. Specifically, the three invasive plants exhibited varied responses to the presence of the 10 native plants. Plant size traits played significant roles in determining the competitive outcomes. Furthermore, disparities in competitive strategies between invasive and native plants may facilitate their co‐occurrence.

### 4.1 The invasive and native plants both determined invasions

Our results demonstrated that the three widely distributed invasive plants did not always exhibit better competitive abilities than the dominant native plants in recipient communities in the Qinling Mountains. In contrast, some native plants in our study exhibited higher competitive abilities when mixed with invasive plants. Our findings and the previous researches all demonstrated that competitive outcomes between native dominant plants and invasive plants may influence invasion. According to our results, *A. argyi*, *A. lavandulifolia*, and *C. album* could prevent invasion of the three invasive plants, and *S. viridis*, *A. vestita*, and *A. annua* could prevent invasion of *E. Canadensis*, *G. quadriradiata*, and *E. annuus* respectively, because that their competitive abilities were improved when the native plants mixed with invasive plants, and their competitive abilities were higher than that of the accompanying invasive plant. These results were consistent with previous studies. *A. argyi*, *A. lavandulifolia*, *A. vestita*, and *A. annua* extracts could inhibit the growth of *G. quadriradiata* and *E. Canadensis* through allelopathy, respectively (Wang et al., 2023). Moreover, as a native plant, *A. argyi* was less affected by environmental changes than invasive plants (Cui et al., 2023). *S. viridis* could be utilized to control the invasion because it exhibited higher competitive ability than the invasive plant, *Ageratina adenophora* (Pan et al., 2022). Moreover, the germination potential and the germination index of *S. viridis* could significantly increase by water extracts from the root and leaf of *Cenchrus incertus*, an invasive plant (Bao et al., 2023). When mixed with invasive plants, *C. album* could increase their specific leaf areas and could increase light utilization efficiency (Guo et al., 2023; Yang et al., 2014). Moreover, *C. album* could reduce the leaf nitrogen content of invasive plant with high nitrogen acquisition capacity under nitrogen deposition (Guo et al., 2023).

Additionally, there were other interspecific relationships in our study: Although native plants took advantages of competition in mixed pots according to interspecific relationship results, the competitive abilities of both native and invasive plant were negative. For instance, combinations of native *A. bidentata* and invasive *G. quadriradiata*, and native *A. dubia* and invasive *E. annuus*, or *E. Canadensis* showcased this trend. The vacated niches resulting from the decline of dominant native plants could be exploited by co‐occurring subdominant invaders, posing ongoing threats to recipient communities (Shen et al., 2023). Therefore, vigilance regarding secondary invasions would become imperative, particularly if the competitive abilities of both invasive and native plants declined when they co‐occurred.

### 4.2 Size traits of invasive and native plants influenced invasion

According to our results, the size of both invasive and native plants had significant effects on invasion. Our results were consistent with those of previous studies demonstrating that plant size was generally regarded as reflecting competitive ability (Chen et al., 2018; Van Kleunen et al., 2010), and was consistently associated with the invasive ability of invasive plants and the resistance ability of native plants. Asymmetric size competition may lead to disproportionate competitive effects (Schwinning & Fox, 1995). Larger plants are expected to experience competitive exclusion by preventing them from meeting their minimum resource requirements (Adler et al., 2013; DeMalach et al., 2016; Hutchings & de Kroon, 1994). For example, taller plants always lead to competitive exclusion by intercepting more light to suppress the growth of shorter plants (Divíšek et al., 2018). Thus, being taller and outcompeting other plants by shading may be the key to the success of invasive plants in temperate ecosystems (Gallagher et al., 2015) and in our experiment.

In addition, plant height also represented the growth rate in our experiments because seedlings germinated from seeds and were sown at the same time. Competitive ability depends on the relative growth rate (Grace, 1990). Fast‐growing species would take a competitive advantage by exploiting and utilizing environmental resources faster (Morris et al., 2011; Richards et al., 2006), which could result in the competitive exclusion of co‐occurring species, leading to successful invasion (Keser et al., 2015). Therefore, our findings illustrated that invasive plant with faster growth rates have the capability to successfully invade.

In contrast to invasive plants, native plants demonstrated a competitive advantage based on diameter and aboveground biomass rather than height, as indicated by the results of the multiple linear regression analysis (Table 1). This could be because there are two functional groups in the native plants, forbs, and grasses, which exhibit different growth forms. For example, as a cespitose grass, *S. sibiricus* gained a competitive advantage for light not only by growing taller, but also by producing more tillers. Thus, diameter and aboveground biomass significantly affected competitive abilities of native plant in our study.

### 4.3 Divergence between invasive plant and native plant facilitated invasion

Our finding supported the phenotypic divergence hypothesis, suggesting that divergence in functional traits contributes to successful invasion. One intriguing observation from our study was the positive correlation between the diameter of native plants and the competitive abilities of invasive plants. This seemed to imply that larger‐diameter native plants could inadvertently support the growth of invasive forbs. This seeming paradox could be elucidated by considering different growth strategies. Plants with larger diameters could obtain growth advantages by acquiring more light through producing more tillers, thereby gaining a competitive advantage. In contrast, invasive forbs could obtain growth advantages by growing taller, effectively minimizing direct competition for resources.

These results highlighted a key aspect of invasive plant success: the ability to exploit different resource acquisition strategies, thus avoiding direct competition with native plants. Such strategic differences in resource utilization underscore the dynamic nature of interspecific relationships between native and invasive plants, ultimately influencing the invasion process. Our findings underscore the importance of understanding these discrepancies in competitive strategies, as they can significantly impact the dynamics of plant invasion within the ecosystem. This lends support to the phenotypic divergence hypothesis, suggesting that invasive plants exhibit dissimilar traits from native plants to effectively disrupt the community (Ferenc & Sheppard, 2020; Ordonez et al., 2010).

## 5 CONCLUSION

The pairwise common garden experiment demonstrated that invasion was determined by both invasive plants and native plants in the Qinling Mountains. Size traits of the 3 invasive plants and the 10 native plants influenced invasion by affecting competitive abilities. Additionally, the divergences in competitive strategies between native and invasive plants facilitated invasion.

Our results also suggested that the three invasive plants, *E. annuus*, *E. canadensis*, and *G. quadriradiata*, maybe impossible to cause invasive hazards in the Qinling Mountains. Simultaneously, our findings demonstrated the potential of using native plants, especially *A. argyi*, *A. lavandulifolia*, and *C. album* to control the invasions of the invasive plants, *E. annuus*, *E. canadensis*, and *G. quadriradiata*. Furthermore, since these native plants in this study are widely distributed in China and Asia, our results also have broad applications. Additionally, the conclusions drawn from this study were based on specific experimental conditions involving only 3 invasive plants and 10 native plants. It is important to acknowledge that the dynamics of plant interactions within natural ecosystems can be considerably more complex. Therefore, further research is needed to explore the effectiveness of different combinations of native plants against alien plant invasion in the future.

## AUTHOR CONTRIBUTIONS

**Yang Li:** Conceptualization (equal); data curation (lead); formal analysis (lead); funding acquisition (equal); investigation (equal); methodology (equal); writing – original draft (lead); writing – review and editing (lead). **Ming Yue:** Conceptualization (equal); methodology (equal); writing – review and editing (equal). **Yuchao Wang:** Conceptualization (equal); data curation (equal); formal analysis (equal); funding acquisition (equal); methodology (equal); writing – review and editing (equal). **Zhuxin Mao:** Data curation (supporting); investigation (equal); writing – original draft (supporting). **Jinlin Lyv:** Data curation (supporting); investigation (equal); writing – original draft (supporting). **Qian Li:** Data curation (supporting); investigation (equal).

## CONFLICT OF INTEREST STATEMENT

The authors declare no competing interests.

## Data Availability

The data that support the findings of this study are openly available in the Dryad Digital Repository at https://doi.org/10.5061/dryad.pvmcvdntp.
